# Neurotransmitters, Cell Types, and Circuit Mechanisms of Motor Skill Learning and Clinical Applications

**DOI:** 10.3389/fneur.2021.616820

**Published:** 2021-02-25

**Authors:** Wotu Tian, Shengdi Chen

**Affiliations:** Department of Neurology and Institute of Neurology, Ruijin Hospital Affiliated to Shanghai Jiao Tong University School of Medicine, Shanghai, China

**Keywords:** motor skill learning, neurotransmitter, neural circuitry, neurodegeneration, Parkinson's disease, Huntington's disease

## Abstract

Animals acquire motor skills to better survive and adapt to a changing environment. The ability to learn novel motor actions without disturbing learned ones is essential to maintaining a broad motor repertoire. During motor learning, the brain makes a series of adjustments to build novel sensory–motor relationships that are stored within specific circuits for long-term retention. The neural mechanism of learning novel motor actions and transforming them into long-term memory still remains unclear. Here we review the latest findings with regard to the contributions of various brain subregions, cell types, and neurotransmitters to motor learning. Aiming to seek therapeutic strategies to restore the motor memory in relative neurodegenerative disorders, we also briefly describe the common experimental tests and manipulations for motor memory in rodents.

## Introduction

Motor learning implies a process of change or improvement in motor action to perform the requested task by practicing and refining (1). There are three components of motor learning: motor skill learning, motor adaptation, and motor action selection (2). Motor skill learning, including motor sequence learning, consists of a series of relatively slow changes in motor functions leading to improved performance. Motor adaptation refers to faster changes in motor behavior that preserve stable performance of learned behavior despite small fluctuations in the environment. Besides the abovementioned motor learning categories, motor action selection is an intermediate one, which is described as the task of choosing which of several possible behaviors to execute (2, 3). In daily life, different categories of motor learning often overlap (4). In this review, we mainly concentrate on motor skill learning. The learning of new skills involves three stages: the initial acquisition phase with fast amelioration in performance, the following consolidation phase with more gradual ameliorations as skills are automatized, and the final retention phase in which the long-lasting memory is formed (5–9). The basal ganglia, cerebellum, and motor cortex are the brain areas involved in motor learning through their circuits (5, 10–12). Motor skill learning is extremely critical for optimizing behavior (13, 14). From a computation-neurobiological perspective, a good motor task should be able to be repeated, emulating a reference model as precisely as possible, aiming to attain the best performance (15). On the other hand, extensive studies have proved that motor skill learning is damaged in patients with Parkinson's disease (PD) (16–20), presymptomatic and symptomatic Huntington's disease (HD) (21–23), and primary dystonia (24). However, the mechanisms on how the brain links various actions together into fluid chains of behavior which are able to be recalled later are still not clear (25). This review addresses the latest findings in motor skill learning, aiming to better comprehend the functional contribution of various brain subregions, cell types, and neurotransmitter systems to this type of memory, evaluate the impact of genetic and pharmacological manipulations, and identify potential treatments for related neurological disorders.

## Common Behavior Tests for Motor Skill Learning in Rodents

In the investigations of motor skill learning, smaller animals like rodents are preferred by investigators due to rapid reproduction and relatively low costs. The single use of one test may not be comprehensive for detecting all aspects of learning dysfunction. In light of this, a few of behavioral tests have been applied to evaluate and quantify the presence of motor skill learning impairments in rodents.

### Rotarod Test

The rotarod test is a frequently used paradigm to measure a rodent's ability to keep itself on a rotating rod at accelerating speeds (26). This requires mice to keep balance on the rod and measures their latency to drop down which generally correlates motor skill learning (26, 27). The speed is gradually accelerated from 0 to 40 rpm over 5 min, and the rodents are tested for several trials a day (28). Motor skill learning can be assessed by repeated daily testing and suggested by the decrease in latency to drop down during sequential testing sessions (29). Most performance improvements occur on the first day of training (30). However, with longitudinally repeated tests, the animals can learn that the consequences of falling are harmless (28). Thus, individual animals may refuse testing and directly fall once they are put on the rod. In this case, we tend to appropriately increase the sample size, so as to remove the individual outliers with extremely abnormal results in statistical analysis.

### Food-Reaching Task

Animals are trained to reach for a food pellet through a narrow slot with a preferred limb (31–33). As a reinforcement learned behavior, it requires acquisition of a skilled reaching movement through daily training over several weeks (33). A successful reach is scored when the animal grasps the food pellet and brings it into the cage and to its mouth without dropping the pellet (34). The basic measures include (a) total number of reach attempts, (b) number of sensory errors, and (c) percentage of successful reaches (33). The shortcoming is that the evaluation is a readout of the learning sequence order rather than focusing on improvements of motor behavior itself (2). After all, the reaching movements themselves are simple without speed-accuracy constraint (35).

### Wheel-Skill Learning

Although developed to measure the animal's voluntary activity in home cage (28), the running wheel is also used to investigate procedural learning (36, 37). At the beginning of the training, rats usually could not run on the wheel without causing it to swing (37). Over continued training sessions, they gradually learn how to adjust their movements on the wheel so as to stabilize it and avoid swinging. Within the first training sessions, rats learn the wheel skill fast and the wheel swings provide a measurement of performance error during skill learning (37). The factors that affect wheel-skill learning include the number of trails in each session and the total amount of training sessions (38). However, it does not depend on motivational manipulations, such as forced locomotion in rotarod, food deprivation in instrumental learning, and electric shocks in avoidance learning (39). It is noteworthy that the running-wheel behavior in female animals could be affected by estrous cycle (40). Thus, mixed-gender cohorts should be avoided in these tests (28).

In addition to the tests described above, there are also other assays being used to assess motor learning, such as the beam-walking test. With several days of training followed by one day of testing, the goal is for the tested rodent to keep balance and walk through a narrow viaduct beam to a safe platform (41). Performance of the subjects, including the time to walk across the beam and the times of paw slips during the test, has been validated as a measure of fine coordination. The beam walking test can be useful especially when assessing balancing capacity and subtle deficits in motor skills which are uneasy to be detected by other tests (41).

## Specific Brain Subregions and Cell Types Involved in Motor Skill Learning

By means of neuroimaging, lesions, electrical stimulation, and electrophysiological recordings, the major brain regions involved in motor skill learning have been disclosed, including primary motor cortex (M1), basal ganglia (BG), and cerebellum. Each region consists of various intermingled cell types connected in specific circuitry and motor skill occurs via changes in neuronal excitability, synaptic strength, and circuit connectivity (42).

### Primary Motor Cortex (M1)

Compared to other nuclei involved in motor learning, M1 acts as a controller which sends commands directly or indirectly to motor neurons (2, 43, 44). The motor cortex provides independent limb control to execute specific actions with high speed and precision and allows flexible synergies of performance related with novel tasks or objects (45, 46). Motor training can induce functional and structural synaptic plasticity in motor map organization (47–50), which is not simply caused by increased use (51). Not like pure exercise or recall of learned skills, a new motor skill learning is able to efficiently trigger the spine formation of pyramidal neurons in the layer V motor cortex (51). Moreover, the tested subjects' performance is closely related with the degree of new spine formation (51–53). In addition, learning-triggered newly formed spines provide a structural basis for enhancing synaptic strength, which are given priority to be stabilized and retained with new skill memory (52, 53). However, longer training could lead to increased spine elimination, indicating that skill refinement might be based on removal of inappropriate connections (52, 53). The skill learning-related spinogenesis could be further induced in the same place where baseline control originates by training the pretrained animals (52, 53). In the sensorimotor cortex, functional synaptic plasticity including long-term depression (LTD) and long-term potentiation (LTP) is the crucial mechanism for acquiring motor skills (54). In humans, improved performance of sequential finger movements are identified to be related with elevation of blood-oxygen level-dependent (BOLD) signal in M1 (55, 56), which is enhanced by transcranial direct current stimulation of M1 (56, 57) and inhibited by repetitive transcranial magnetic stimulation (TMS) of M1 (10). Moreover, TMS could induce piano-playing behaviors in pianists but not in controls, suggesting that M1 can encode novel skills via continuous practicing (58). In rats, the skill acquisition can be completely abolished by destroying dopaminergic (DAergic) projections to the motor cortex, demonstrating that skill learning needs to occur in M1 directly (59).

### Basal Ganglia (BG)

The BG, preponderantly involved in movement control and skill learning (60–66), are highly conserved in both anatomy and neurotransmitter localization that consist of cortico-striato-pallido-thalamocortical loops (67). In rodents, BG circuits are critical in task improvement through promoting execution quality. The tested mice's improvement of their performance in rotarod is correlated with synaptic strength enhancement in the striatum (6, 68). In the food reaching task, protein synthesis inhibition in the striatum could impair early stages of learning in rats (69).

The striatum is the main input nucleus of the BG. It works as the central meeting point which compiles and integrates the information from the thalamus, the cortex, and the midbrain DAergic innervation before processing of motor output (70–74). The ventral striatum is involved in reward-related learning due to its anatomical connection with limbic structures (75–78). The dorsal striatum gets involved in movement and action selection, and it mainly receives innervation from the substantia nigra (SN) and cortex (79–84). For the direct pathway, the net effect of activating D1-expressing medium spiny neurons (MSNs) is facilitation of movement by disinhibiting neurons in the motor cortex (85). For the indirect pathway, the net effect of activating D2-expressing MSNs is suppression of movement by inhibiting neurons in the motor cortex (85). When dopamine (DA) is released from DAergic neurons of the substantia nigra pars compacta (SNc) to the dorsal striatum, the direct and indirect pathways are enhanced and attenuated respectively, and vice versa (25). To be noted, the cortical inputs to the BG are unevenly distributed across the two pathways, with the indirect pathway receiving more from the motor cortex and the direct pathway receiving more from somatosensory and limbic systems (74). Within the dorsal striatum, the medial and lateral parts also play various roles in instrumental learning (25, 86). In the rotarod task, improvement of early stage (action selection) depends on striatal projection to the prefrontal cortex (6, 8), while improvement across training days (execution of sequence elements) depends on striatal projection to the sensorimotor cortex (6). In the BG, the motor functions are closely related with non-motor functions. For instance, most of striatal neurons are involved in both reward- and movement-related activities through combining both reward information and motor actions to obtain the reward (87–89). A neuronal system showing such property usually indicates its role in habit learning and goal-directed behavior (89–91).

### Cerebellum

From the phylogenetic perspective, the cerebellum is a highly conserved brain architecture across all the vertebrates (92, 93), indicating a sustained evolutionary requirement for a specific computation ability (2). The cerebellum is necessary for adaptation of eye and limb movements, which is engaged in finetuning movement and learning novel motor tasks in real-time (94), through its feed-forward structures from parallel fibers to Purkinje cells, which inhibit the inferior olive and the deep nuclei of the cerebellum (15). The cerebellum is believed to be a site of supervised learning, aiming to adjust the movement pattern by using feedback from the system and further improve future performance (95). Generally, our procedural memories formed in the cerebellum exhibit at least two types of information coding: rate coding and temporal coding (96–99). In the cerebellum, different coding schemes are used within different modules to produce and express various memories. For example, zebrin-negative zones predominantly form the memories by inhibition mechanisms and express the memories partially by temporal coding. While zebrin-positive zones mainly form the memories by enhancement mechanisms and express the memories by rate coding (100). The rotarod performance can be damaged by inhibiting the LTD at parallel fiber-Purkinje cells in the cerebellum (101). The cortico-BG-thalamo-cortical loop is essential for skilled motor coordination, and the LTD in cerebellum plays a role in movement optimization for environmental conditions (102).

Differential cortical and subcortical regions activated by long (days to weeks) (5) and short (minutes to hours) (103, 104) times of motor learning have been shown by numerous functional studies. Among all these brain areas, M1 is a critical structure for skill execution but it is still in the location for stereotypy which is learned initially through BG dependent processes (2). During the motor skill learning process, BG is likely to infuse variability for exploration and then when the best performance matures, variability is decreased and stereotypy and automatization arise (105). The cerebellum controls fine motor skills as well as motor adaptation and coordination (95).

## Neurotransmitters and Neuromodulators

A huge emphasis has been put into new methodologies for precise cellular localization of neurotransmitters and neuromodulators, enzymes involved in their synthesis or degradation, receptors, and transporters, which markedly improved our understanding of the molecular pathways that govern motor skill learning. In this section, we review current progress on the mechanisms by which differential modulators get involved in a pathway-specific manner during motor skill learning.

### Dopaminergic System

DA functions by binding to its receptors, which are a group of G protein-coupled receptors (GPCR) and function through the second-messenger system (106, 107). In the primary motor cortex, inhibition of either D1 or D2 receptors could impair skill acquisition (108). In the motor cortex, D1 and D2 receptors play different roles in the regulation of synaptic plasticity: predominantly modulate spine elimination and spine formation, respectively (51). As mentioned above, there are two distinct pathways for DAergic modulation of primary motor cortex including (1) mesocortical projections: directly project from VTA and SNc to directly modulate primary motor cortex (108–110) and (2) nigrostriatal projections: activate a set of BG nuclei and indirectly modulate the primary motor cortex (111, 112).

In physiological conditions, DA exerts an irreplaceable effect in regulating bidirectional plasticity of MSNs (113). For the striatal MSNs, the spine density is significantly decreased in the absence of DA by means of 6-hydroxydopamine lesion of the medial forebrain bundle (MFB) (114). In the motor cortex of various mouse models of PD, abnormal remodeling of neuronal circuits has been disclosed by 2-photon *in vivo* imaging microscopy (115). DA is required for the formation of LTP, which likely is a fingerprint mechanism of a motor memory trace within M1 (116). In 1-methyl-4-phenyl-1,2,3,6-tetrahydropyridine (MPTP)-injected mice, DA depletion leads to marked instability of synaptic connections and significant spine remodeling in the motor cortex (115). In such mice, motor learning-induced newly formed spines failed to stay stable and were eliminated then, which is associated with impaired retainment of motor memory in PD (115). Although motor symptoms of PD can be alleviated by levodopa (L-dopa) treatment, it was unable to ameliorate functional plasticity in the motor cortex (117), as well as motor skill learning (118).

### Cholinergic System

The cholinergic system is correlated with a wide range of neural processes, such as motor, attention, learning, and memory functions (119). In the striatum, acetylcholine (ACh) mainly arises from cholinergic interneurons (CINs), which is involved in controlling the late component of the motor skill learning (120, 121). The cholinergic inputs are activated by ACh binding to nicotinic receptors (nAChRs) on the DAergic axons (122). Thus, the striatum DA release can be directly triggered by CINs tonic firing, independent of the activity of DAergic neuron activity (123, 124). However, in the absence of ACh, DA release is found to be proportional to the firing rate of DAergic neurons (122). Meanwhile, the computational modeling study of PD showed that the lower DA concentration in turn leads to shortening of CIN activity pause in the striatum, and the phasic DA excursion drives learning (125).

In the primary motor cortex, the diffuse cholinergic afferents regulate the synaptic efficiency of horizontal connections (126). Blocking cholinoreceptors is able to alter the learned reaching task in rats (127). Another study of rat showed that increase in ACh levels during early sleep prevented motor memory consolidation in experiments with physostigmine (128). The role of cholinergic connections in motor cortex plasticity also highlights how inhibition of interfering coordinations forms when new movements are learned (129).

### Endocannabinoid (eCB) System

In the brain, cannabinoid receptor 1 (CB1) abundantly distribute across the cerebellum, cortical layers I and IV, BG, CA1 pyramidal cell layer, and dentate gyrus (130). Cannabinoid receptor 2 (CB2) was later identified to be highly expressed in the immune system (131, 132). Then, CB1 and CB2 could be activated by the lipids anandamide and 2-arachidonoyl-glycerol (2-AG) with high affinity and efficacy in the brain and intestinal system, which were named eCBs (133–135). The eCB system also includes enzymes involved in eCB biosynthesis and inactivation. The biosynthesis of 2-AG and other monoacylglycerols is catalyzed by diacylglycerol lipase α and β (DAGLα, DAGLβ) (136). The hydrolysis of 2-AG and other monoacylglycerols is catalyzed by monoacylglycerol lipase (MAGL) (137). CB1 is predominantly located in presynaptical membranes of inhibitory and excitatory neurons, which can suppress vesicular release of gamma-aminobutyric acid (GABA) or glutamate and voltage-gated Ca^2+^ channels in a feedback way (138, 139). In addition, DAGLα is located in postsynaptic membranes and MAGL is located in axon terminals (138). It is suggested that eCBs, especially 2-AG, are inhibitory retrograde neuromodulators (140).

The endocannabinoids, acting as retrograde messengers, are critical for fine-tuning neuronal excitability and synaptic plasticity and involved in neurobiological mechanisms underlying mood, perception, cognition, locomotion, reward-seeking, and motivation-processing (141–144). The cannabinoids have been found with neuroprotective functions in animal models of stroke, epilepsy, HD, PD, multiple sclerosis, and Alzheimer disease (145–150). Biphasic dysregulation of CB1 was disclosed in different PD animal models: hypoactivity at presymptomatic/early stages and hyperactivity at later stages (151–153). The key influence of eCBs in motor behavior, especially in motor learning, has been highlighted (94, 154). The mice without CB1 receptors show less voluntary running behavior in a housed running wheel than wild-type littermates (155). It is suggested that CB1 receptors control the running behavior rather than the locomotor behavior (156, 157). CB1 knockout mice were demonstrated with impairment of cerebellum-dependent discrete motor learning (158). In turn, the motor skill training was found to rescue nicotine-induced damage of synaptic plasticity mediated by eCB in the dorsolateral striatum (159).

### GABA and Glutamate

In the BG circuitry, the strength of glutamatergic neurons is dynamically adjusted through long-term plasticity, which regulates motor function and information flow within the BG network (160). In the BG, long-term plasticity of glutamatergic synapses is an essential contributor to adapted motor execution, among which LTD is the most common form of synaptic adaptation (160). Ninety five percent of striatal neurons are MSNs, including direct-pathway MSNs (dMSNs) and indirect-pathway MSNs (iMSNs): (I) dMSNs expressing dynorphin and substance P bear M4 muscarinic and D1 receptors on the membrane and send projections to the BG output structures and (II) iMSNs expressing enkephalin bear adenosine 2A (A2A) and D2 receptors and send projections to GPe (161–163). In the striatum, LTD exerts effects on the postsynaptic activation of metabotropic glutamate receptors (mGluR), leading to eCB production and CB1-mediated reduction of presynaptic glutamate release. Meanwhile, SNr-LTD depends on N-methyl-D-aspartic acid receptor (NMDAR)-triggered endocytosis of postsynaptic α-amino-3-hydroxy-5-methyl-4-isoxazolepropionic acid receptor (AMPAR) and is independent of mGluR and eCB (160).

A magnetic resonance spectroscopy (MRS) study in human showed that motor sequence learning is associated with a reduced GABA concentration in M1 motor cortex (164, 165). In rat, motor learning-dependent plasticity was found to be regulated by AMPA/GABA receptors of the horizontal connection layer II/III in M1 (102). Motor training could rapid eliminate inhibitory boutons of distal dendrites in layer II/III (166). Specifically, an immediate decrease of axonal boutons occurred on somatostatin (SST) expressing GABAergic neurons after motor training began (166).

**Figure 1:**
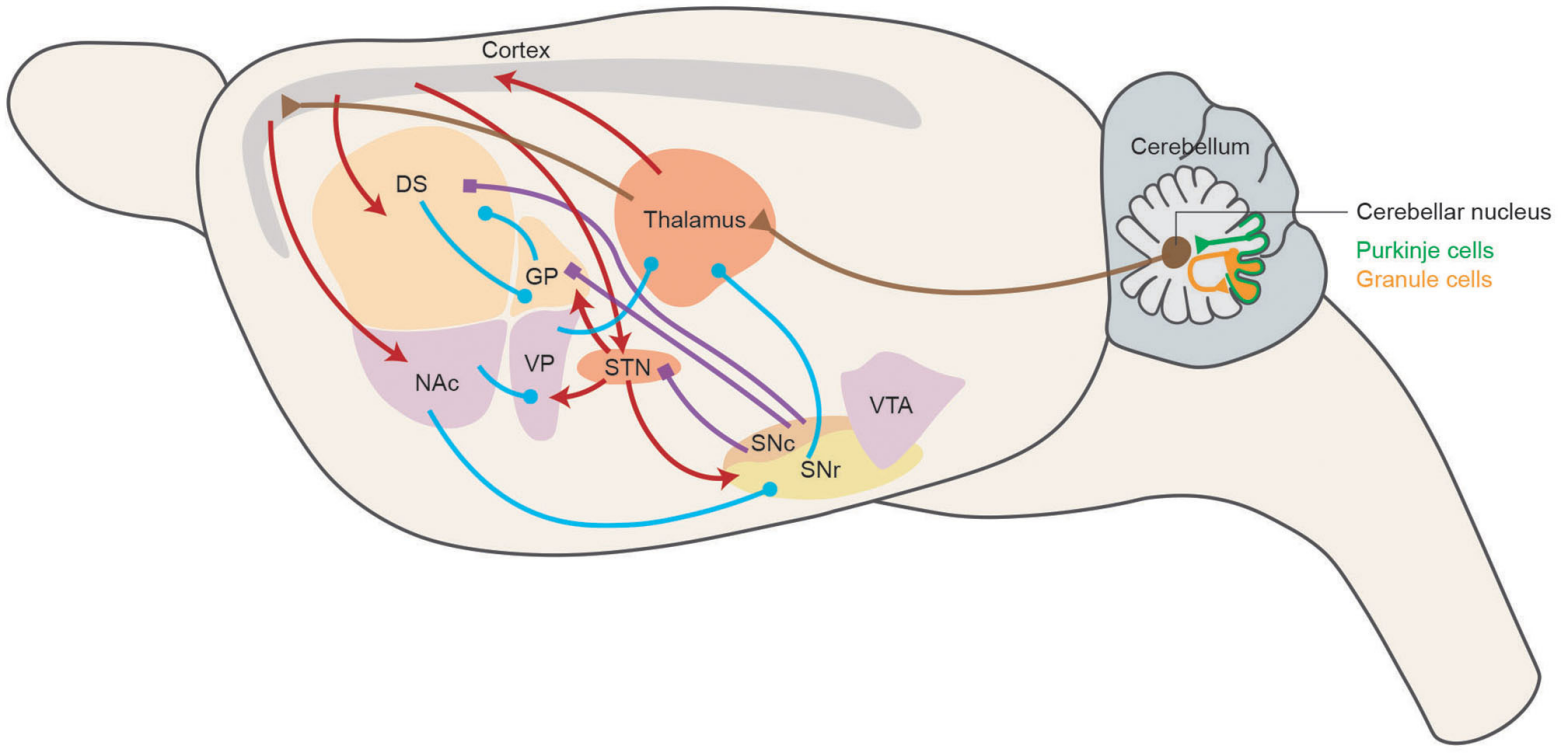
Motor skill learning circuitry in rodents. Schematic representing the main connections of the basal ganglia network in a sagittal section of the rodent brain. Dopaminergic, GABAergic, and glutamatergic projections are depicted in blue, purple, and red, respectively. Cerebellar related circuitry is in brown. DS, dorsal striatum; NAc, nucleus accumbens; GP, globus pallidus; VP, ventral pallidum; STN, subthalamic nucleus; SNc, substantia nigra pars compacta; SNr, substantia nigra pars reticulata; VTA, ventral tegmental area.

In summarization, motor skill learning is the integrative result of different neuromodulator systems, among which every part contributes to a different aspect of learning. Different neural mechanisms check and balance each other to acquire and store motor skills efficiently. The circuitries involved in motor skill learning are summarized in Figure 1, representing the main connections and neuromodulator systems among BG, M1, and cerebellum of rodent brain in a sagittal diagram.

## Genetic Manipulations

Optogenetics and chemogenetics are now widely used circuit-based techniques to acutely and reversibly suppress or activate cell-type-specific neuronal firing activity through the use of a genetically mediated actuator expressed on the cell membrane. Here we introduce the mechanisms and applications of major genetic manipulations in exploring specific brain areas and cell types related with motor skill learning.

### Optogenetics

Optogenetics, by using genetically encodable light-activated proteins, allows for cell-type (167–169) and projection-specific (170, 171) manipulation of neural circuit elements with precise temporal control. In neural systems, the most commonly used are the channelrhodopsins (ChR2, ChR1, VChR1, and SFOs) to excite neurons, as well as archaerhodopsin-3 (Arch) and enhanced halorhodopsin (eNpHR2.0 and eNpHR3.0) to inhibit neuronal activity (168, 172–174). Within the striatum, optogenetics helped characterize the inhibition of MSNs by CINs as well as confirm the opposing relationship between direct and indirect pathway MSNs (175). In the dorsal striatum, through expressing ChR2 in iMSNs and dMSNs, activation of dMSNs increased locomotion and reduced freezing, while activation of iMSNs induced freezing gait, bradykinesia, and hindered locomotor initiations (176). Using a similar method, dMSNs and iMSNs of the dorsal striatum also showed opposing influences on reinforcement (177). In the mouse cerebellum, the memory of oculomotor learning could be artificially implanted by optogenetic stimulation of the Purkinje cells or the climbing fibers (178). Optogenetic suppressions of different brain regions at different stages of skill training enable us to better understand when and how each region gets involved into learning: (1) primary visual cortex (V1) suppression could reduce accuracy across all training stages; (2) anterior cingulate cortex (ACC) suppression decreased accuracy during learning; and (3) hippocampus suppression affected learning more mildly (179). The combination of optogenetics, *in vivo* imaging, and pharmacological manipulations revealed that sensory experience transduced through the granule neuron pathway could orchestrate motor learning through remodeling chromatin architecture and neural circuit activity in the anterior dorsal cerebellar vermis of mouse brain (180).

### Chemogenetics

By means of mall molecules that activate engineered receptors targeting to specific cell types, genetically encoded neuron manipulation tools have been developed to remotely control diverse neuronal/non-neuronal functions (181). Early chemogenetic technologies were based on GPCRs (182). According to the downstream effector system initiated, GPCRs could suppress or excite neuronal firing (183). However, these early-generation tools have not been broadly adopted *in vivo* studies due to the relatively weak potency of synthetic ligands (184) and adenosine (185–187) or given modest signaling (188). To overcome these problems, a new platform called DREADD (designer receptor exclusively activated by designer drug) was developed (189), which uniquely get activated by inert molecule and influence neural processes (190, 191). The most commonly used DREADD receptors include the human muscarinic excitatory and inhibitory receptors (hM3Dq and hM4Di), which can be activated by clozapine-N-oxide (CNO) or low concentration of clozapine (CLZ) (191, 192). In the lever-pushing learning paradigm, by combining chemogenetics and two-photon imaging, mice were trained to perform the task in response to a sound cue, followed by monitoring striatal neuron activity. It helped distinguish that D1 neuron silencing impaired initiation of the learned motor, while D2 neuron silencing increased false performance of lever pushing (193). In the MPTP-injection mouse model, chemogenetic re-activation of SST inhibitory interneurons could alleviate the structural and functional deficits of dendritic spines, as well as enhance rotarod learning (194).

To be noted, it is critical to set stringent experimental controls, because even a slight alteration in designing behavior tests or in choosing DREADD receptor or opsin can make cross-study comparisons difficult (195). Therefore, it is strongly emphasized to study the replication and pay attention to the reported technical challenges or negative findings.

## Pharmacological Manipulations

### 6-Hydroxydopamine (6-OHDA)

6-OHDA, also known as oxidopamine or 2,4,5-trihydroxyphenethylamine, is a catecholamine neurotoxin used to destroy DAergic and noradrenergic neurons in the brain (196). Although there are different techniques for DA depletion, the most commonly employed way is to inject 6-OHDA into the striatum or into MFB (33). Intrastriatal injections consist of four infusions of 6-OHDA spanning the entire length of the striatum. This induces direct toxic damage to the DAergic axon terminals and gradual DA depletion occurs over 4 weeks (197). However, MFB injection involves one infusion into the DAergic projections from the SN to striatum and DA depletion and Parkinson's symptoms occur more rapidly and usually within 48 h. The degree of DA depletion can be verified by using immunostaining to assess the levels of tyrosine hydroxylase (TH) (197). Previous studies have demonstrated impaired rotarod behavior in rats with 6-OHDA lesion of striatum during the pre-motor stage of PD (197, 198).

### 1-Methyl-4-phenyl-1,2,3,6-Tetrahydropyridine (MPTP)

MPTP is a highly lipophilic compound and easy to cross the blood–brain barrier. In the brain, under the catalysis of enzyme monoamine oxidase-B (MAO-B), it is converted to the active metabolite, 1-methyl-4-phenylpyridinium (MPP^+^) (199). A series of cytotoxic mechanisms leading to apoptotic cell death are induced by MPP^+^, such as oxidative stress, mitochondrial dysfunction, and energetic failure (199). MPP^+^ induces vesicular DA into the cytoplasm, leading to the production of cytotoxic substances (200). MPTP-injected primate model, manifesting profound parkinsonian syndrome, has been widely used for development of novel therapeutics of PD (201). However, MPTP has been less used in rats due to the absence of MAO-B leading to limited toxicity of MPTP in rat brain. Conversely, MPTP does produce obvious DA depletion in mice through downregulating the activity of TH in the biosynthetic pathway for catecholamines in DAergic neurons (201).

### Rotenone

Rotenone is an insecticide and piscicide that has been related to a high risk of PD (202, 203). Rotenone impairs mitochondrial transport and abolishes the potentiation of the synapse by inhibiting mitochondrial electron transport chain complex I and inducing mitochondrial reactive oxygen species (ROS) generation (204, 205). Rotenone also inhibits microtubule formation from tubulin (206–208). Chronic administration of rotenone could induce a dose- and time-dependent nigro-striatal degeneration by oral administration for mice or intravenous or s.c. infusion for rats (209–212). The administration of rotenone can impair motor behavior, learning, and memory functions in animal model (213–215). In the rotarod test, rotenone-infused rats showed a significantly decreased balancing ability with an increased falling frequency in comparison with control group (216).

## How to Restore Motor Learning and Clinical Application

### DAergic Enhancement

The DAergic system, critical for motor learning, experiences a parallel decline even with normal aging (217–219). This age-dependent decline, contributing to the faded learning ability, involves DA metabolism, receptors, and transporters (219–221). Thus, pharmacologic strategies that enhance DAergic neurotransmission have been tried in patients with motor learning deficiency during stroke recovery and have been proven a promising adjuvant therapy in motor rehabilitation (222–224). In animal studies, DA and DA-receptor agonists have been proved with a positive role in synaptic plasticity, recovery, and learning after brain lesions (220, 225, 226). Experimental studies in healthy humans showed that premedication with L-dopa (precursor of DA) (227) and cabergoline (D2R agonist) (228) improved the elementary motor memory formation (228). The deficiency in motor skill learning in the PITx3^(−/−)^ mice, a commonly used DA deficiency model (229–231), could be rescued with levodopa treatment (232). However, the PITx3^(−/−)^ mice showed a gradual deterioration after cessation of L-dopa treatment (232). Although the clinical strategies of alleviating DA-related symptoms in PD by DAergic replacement have been proved highly successful in treating the motor symptoms (233), the effects on motor learning ability remained controversial probably due to different motor tasks being used. One clinical trial on the effect of L-dopa on patients with mild-moderate PD showed improved learning of upper extremity task (234). Another two clinical trials suggested that the L-dopa medication did not significantly alter learning performance of the stepping task in PD patients (235, 236). However, some studies hold that, since exogenous DA is delivered systemically, it may suppress the striatal activation during the acquisition stage of motor learning (237–239). Moreover, long-term administration of L-dopa could lead to L-dopa-induced dyskinesia in advanced stage of PD (240–242).

### Deep Brain Stimulation (DBS)

Deep brain stimulation (DBS) is the gold standard for surgical treatment in PD patients by modulating specific neural pathways (243). Recently, a clinical trial on PD patients engaged in a visuomotor tracking task disclosed that the impaired sequence motor learning in PD could be partially restored through subthalamic nucleus (STN)-DBS (4). Actually, the disynaptic connections between the cerebellum and BG have been proved in nonhuman primates by viral tracing (244). STN output projects to the ipsilateral cerebellum through pontine synapse and dentate projections form the thalamo-striatal circuitry. Another clinical study in PD showed an obvious positive association of functional connectivity between cerebellar and DBS contacts during STN-DBS. In addition, the PD patients treated with STN-DBS showed the significant learning-related spatial covariance pattern including increased activity in the para-hippocampal gyrus, dorsal premotor cortex, and lateral cerebellum, with covarying reduction in the orbitofrontal cortex and supplementary motor area (SMA) (236). It is suggested that the pathological STN activities could interfere cerebellar functions due to higher firing rate and lacking desynchronization, whereas the electrical stimulation of DBS could liberate or decorrelate the cerebellum from abnormal BG input (245).

### Neurofeedback (NFB) Training

NFB works as a biofeedback technology. In NFB, by displaying the sensory signals (reflecting real-time neural activity) to subjects, they can learn to modulate activity in targeted neural areas involved in specific behaviors or brain functions (246, 247). By using functional magnetic resonance imaging (fMRI), researchers are able to monitor the task-induced changes in neural activation and provide neural signal feedback to the participant in a real-time way (rt-fMRI-NFB) (246, 248). In stroke, PD, and Huntington's disease, rt-fMRI-NFB was proved to alter neural activity in motor-associated areas and to modify specific motor behaviors after the self-regulation training was completed (249–251). A clinical study of PD patients of early stage with NFB training showed an improvement in motor speed during tasks as well as activation in STN and GP, which are connected to SMA (252). This model is consistent with a motor learning study in a healthy population, where the functional coupling between BG and SMA increased with practice (253). The increased activation of SMA could raise the input to STN and the activity of GPi, leading to a changed neural activation pattern within the BG network and thus causing an improvement of symptoms (252).

### Physical Exercise

Given that physical exercise leads to synaptic reorganization and neuroplasticity changes in the corresponding motor cortex (52, 53). Such exercises usually need specially designed movement patterns, which should consider multiple key factors, such as the visual and other external cues, compliance, and attrition of the patients (254). There are many types of exercise which can be grouped into “motor-skill” exercises, such as various kinds of dancing or Tai Chi, which require participants to keep learning and training of novel movements (254, 255). It seems reasonable that sustained “motor skill” exercises may be more beneficial for PD patients (51). In addition, patients diagnosed with Alzheimer's disease taking part into a waltz dance training were significantly improved with procedural learning (256). To be noted, extra auditory cues could be provided by the music during these physical exercises that access the motor cortex via the cerebellum and SMA via the thalamus, causing improvement of gait speed, initiation, coordination, and cadence (254). There is no doubt about the clear benefits to physical exercises, including the increase of endurance, strength, and balance stability. However, the current overall level of evidence for studies in human beings with neurodegenerative disease is still very low, and thus much remains to be known regarding the mechanisms of exercise-mediated relief of motor symptoms (257).

## Conclusion

Here we briefly reviewed the current discoveries of motor learning across rodent and clinical studies on the basis of neural circuitries and neurotransmitter systems in M1, BG, and cerebellum involved in motor learning. In the past few decades, the exploration of the mechanism underneath motor learning has never stopped and kept guiding us better comprehend how the motor memories are formed, stored, recalled, faded, or disturbed as well as restored. With the development of neuro-computational and neuroimaging technologies, along with the combination of genetic and pharmacological manipulations, we could see more essence through the surface than ever. Yet, we believe that more work combining the theoretical progress in rodent models, the use of well-controlled experiments of *in vivo* neuroimaging, and the newly discovered biomarkers for neuron subtypes by genomics and proteomics methods helps to understand the precise nature and the determinants of specific roles played by precisely-defined cell subtypes when spatiotemporally participating into this form of memory.

## Author Contributions

WT contributed by writing the manuscript and creating the figure. SC contributed to the conceptualization and critical review of the manuscript. Both authors contributed equally to drafting the manuscript.

## Conflict of Interest

The authors declare that the research was conducted in the absence of any commercial or financial relationships that could be construed as a potential conflict of interest.
